# Paper-based in vitro tissue chip for delivering programmed mechanical stimuli of local compression and shear flow

**DOI:** 10.1186/s13036-020-00242-5

**Published:** 2020-07-28

**Authors:** Kattika Kaarj, Marianne Madias, Patarajarin Akarapipad, Soohee Cho, Jeong-Yeol Yoon

**Affiliations:** 1 Department of Biosystems Engineering, The University of Arizona, Tucson, AZ USA; 2 Department of Biomedical Engineering, The University of Arizona, Tucson, AZ USA

**Keywords:** Automated flow control, Microcontroller, Paper-based cell culture, Vascular endothelial cell, Cell migration

## Abstract

**Abstract:**

Mechanical stimuli play important roles on the growth, development, and behavior of tissue. A simple and novel paper-based in vitro tissue chip was developed that can deliver two types of mechanical stimuli—local compression and shear flow—in a programmed manner. Rat vascular endothelial cells (RVECs) were patterned on collagen-coated nitrocellulose paper to create a tissue chip. Localized compression and shear flow were introduced by simply tapping and bending the paper chip in a programmed manner, utilizing an inexpensive servo motor controlled by an Arduino microcontroller and powered by batteries. All electrical compartments and a paper-based tissue chip were enclosed in a single 3D-printed enclosure, allowing the whole device to be independently placed within an incubator. This simple device effectively simulated in vivo conditions and induced successful RVEC migration in as early as 5 h. The developed device provides an inexpensive and flexible alternative for delivering mechanical stimuli to other in vitro tissue models.

**Graphical abstract:**

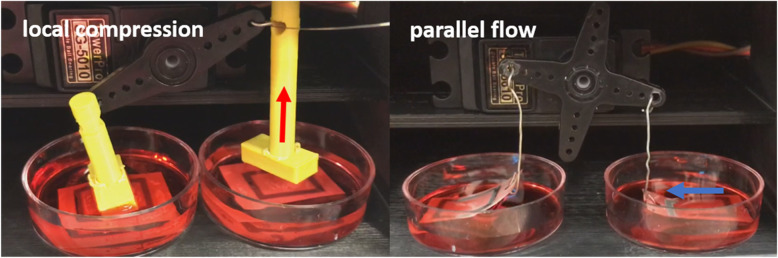

## Introduction

Mechanical stimuli are key parameters for reproducing cellular microenvironment in vitro [1]. The most common mechanical stimuli found in the human body are shear flow, compression, and stretch/strain [2]. Among these stimuli, shear flow has extensively been demonstrated in in vitro tissue models, as it is a fundamental mechanism for delivering medium and solutions of interest throughout tissues and organs. In addition, shear flow can easily be applied to microfluidic tissue models, where the microfluidic channels mimic the network of vessels found in human tissues and organs. Traditionally, the flow control in microfluidic tissue models can be classified into three categories: external pumping (66%), internal pumping (15%), and passive delivery (19%) [3].

The most popular and simplest method is external pumping, typically utilizing a syringe pump or a peristaltic pump. Such external pumping has frequently been demonstrated in various silicone-based microfluidic tissue models to evaluate the effects of shear flow within the model [4–6]. However, those pumps are still bulky in size, expensive, and typically require a separate AC power. To overcome these limitations, a pumping mechanism or component was integrated within microfluidic tissue models, i.e., internal pumping [7, 8]. However, the integration of a pump into a microfluidic device is quite challenging and not at all appropriate for the long-term operation necessary for in vitro tissue models (at least several hours and often several days). As a low-cost and simpler alternative to these external and internal pumping methods, passive flow control has recently emerged, specifically in the past three years. For example, microtiter plate-based tissue models were tilted to generate passive flows, where neuron tissues were used to investigate stem cell differentiation or neurite outgrowth [9, 10]. Hydrostatic pressure was also used to generate the passive flow for studying angiogenesis (formation of new blood vessels from pre-existing vessels) on a vasculature tissue model [11]. While passive flow controls are gaining popularity in recent years, the precise control of flow rate can potentially be challenging, and the platform may fail (typically by leaking) in long-term experiments [12].

Besides shear flow, compression is also an important mechanical stimulus in many tissues including heart, bone, and blood vessels [13–15]. Such compression has typically been demonstrated using pistons on 3D, gel-based cell culture models [13], which can be bulky and costly. Compression has rarely been demonstrated on microfluidic tissue models. In fact, shear flow and compression stimuli require totally different tissue model platforms: shear flow has typically been demonstrated on silicone-based microfluidic tissue models, while compression on 3D gel-based cell culture models. However, some tissues are exposed to both types of mechanical stimuli, e.g. vascular endothelial cells experience both shear flow and compression [16]. Application of stretch and/or strain to in vitro tissue models also requires 3D gel-based cell culture models that are stretchable and/or compressible, which is inappropriate for silicone-based microfluidic models.

**Fig. 1:**
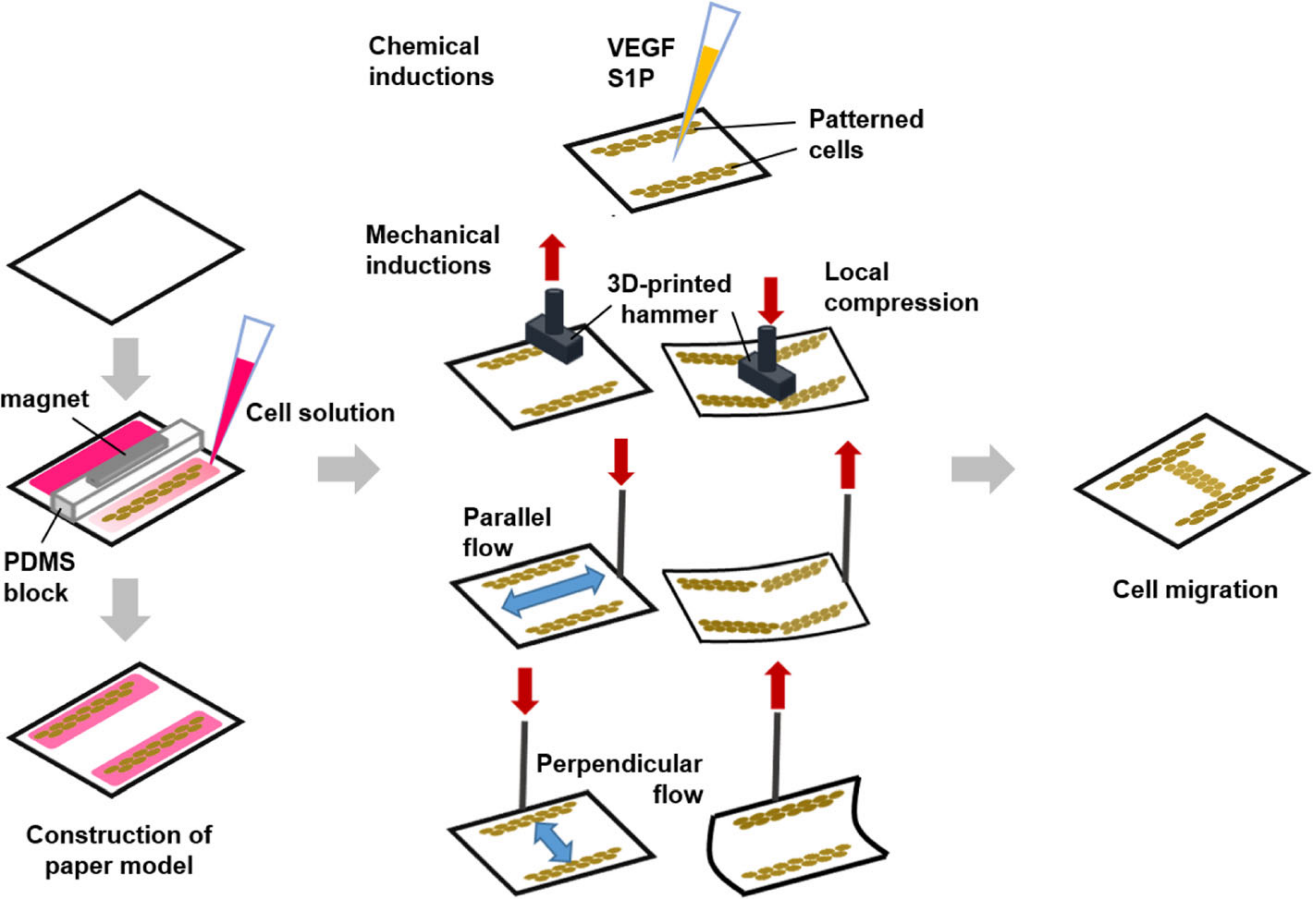
Graphical illustrations of the construction of the paper chip (left), the application of various mechanical stimuli (center), and the resulting cell migration (right)

In this work, we propose a novel, simple, small, and cost-effective device, where both compression and shear flow can be applied to a tissue model in a programmed manner (Fig. 1). This device was tested for a vasculature tissue model, to evaluate the effects of compression and shear flow towards the migration of endothelial cells, i.e. the initial stage of angiogenesis which involved in normal tissue development as well as disease progression. The inhibition, induction, or normalization of angiogenesis has been widely investigated for potential therapeutic strategies [17]. Specifically, tumor growth utilizes angiogenesis to create a network of blood vessels that surround tumor tissue, supplying nutrients and oxygen, and removing waste. Angiogenesis also plays an important role in metastasis, the migration of cancer cells to secondary tissue [18]. Therefore, the inhibition of angiogenesis is a widely accepted therapy for cancer, depriving nutrients and oxygen, and subsequently hindering the progression of metastasis.

Various materials, for example, hydrogel and paper-biomaterial hybrid, have been used as substrates for 3D cell culture, together with mechanical cues adjunction, to create an appropriate microenvironment [19]. The mechanical property of hydrogel can be modulated by adjusting its compositions; however, it still lacks capability to provide the in vivo-like spatiotemporal physical cues mimicking the complexity and heterogeneity of native tissue [20]. On the other hand, papers can provide an ECM-like fibrous structure and porosity. Additionally, papers are flexible and can be easily modified (e.g., cut, fold, create hydrophobic pattern, and even used as a support structure for hydrogel), inexpensive, and applied with the flow referred to as paper-based microfluidic technology [21–23]. In this work, the paper coated with collagen was used as a “flexible” substrate for cell culture, effectively delivering both compression and shear flow on a single platform within our device. Both types of mechanical stimuli could be applied by simply tapping and bending the paper substrate (not possible with the silicone-based microfluidic models). Both mechanical stimuli were delivered to this cell model in a programmed manner using an Arduino microcontroller using an original code, and a servo motor. All components were enclosed in a small, 3D-printed housing, including batteries. To the best of our knowledge, there are no currently published studies demonstrating vascular endothelial cell migration in response to both types of mechanical stimuli on a single platform.

Under the influence of stimuli (e.g., mechanical stimuli, chemical stimuli, and micropatterning structure), cells can polarize and migrate to a certain direction [24]. Endothelial cells were easily patterned directly on a paper substrate, which offered the gel-like environment, enabling fast and affordable patterning and fabrication. Cells were initially seeded and patterned on the peripheral sides of the collagen-coated nitrocellulose paper, without using lithographic or wax printing methods. Paper type and coating material were optimized for successful cell patterning. This patterning mimicked pre-existing capillary vessels, and was subjected to local compression or pulsatile flow through tapping and bending paper substrate under optimized incubation time (supplementary MOV files are included in the supplementary information (Additional files 1 and 2). Cells were free to migrate in between these two patterns. Mechanical stimuli were also accompanied by chemical induction through applying vascular endothelial growth factor (VEGF) to the center area between two cell patterns, or by tumor induction where one of the patterns was seeded with human breast cancer cells. The developed device and findings of this study may inspire innovative strategies in delivering multiple types of mechanical stimuli to in vitro cell models in a programmed manner.

**Additional file 1** Video clip showing the pulsatile local compression (18.5 times/min) applied to the paper chip, as controlled by a servo motor and an Arduino microcontroller.

**Additional file 2** Video clip showing the pulsatile parallel flow (7 cm/s) applied to the paper chip, as controlled by a servo motor and an Arduino microcontroller.

## Results and discussion

### Optimization of paper type, coating, and assay time

**Fig. 2:**
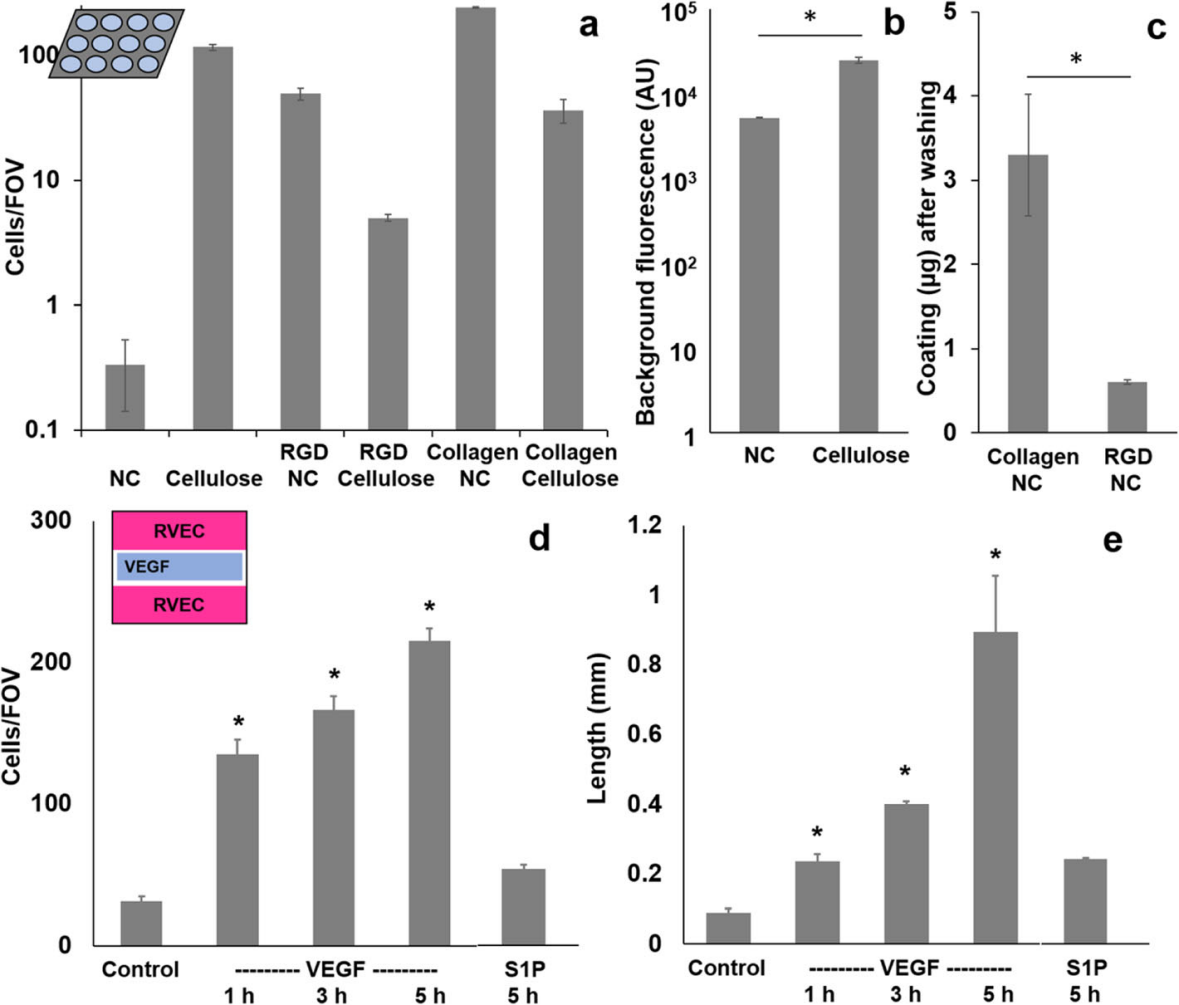
Optimization of the type of paper, coating materials on paper, and chemical stimuli. **a** Average numbers of RVECs on the field-of-view (FOV) on various paper types and coatings, hole-punched into 5-mm diameter circles and cultured on a 96-well plate. **b** Background fluorescence intensities of two types of paper substrates. **c** Amount of coating materials on NC paper after washing. **d**-**e** Average numbers of migrating cells and the length of migration on the RVEC chip under static condition, with VEGF or S1P added to the central area of each chip. Averages of 3 experiments, each from 6 different images of a different paper substrate or a paper chip. Error bars represent standard errors. * represents statistical difference with *p* < 0.05 between two data sets (in b and c) or from control (in d and e)

While paper fibers offer a 3D, extracellular matrix (ECM)-like microenvironment, adhesion of endothelial cells on paper fibers is poor due to the electrostatic repulsion between the paper’s negative charge and the cell membrane’s phosphate groups. This requires the optimization of paper types (cellulose vs. nitrocellulose = NC) and coatings (RGD-containing peptide vs. collagen). RGD peptide sequence should promote cell adhesion through focal adhesion [25]. Collagen-coated NC paper shows a significantly higher number of cells adhered on paper than GRGDSPK-coated NC paper, bare NC paper, and cellulose paper (GRGDSPK-coated, collagen-coated, and bare cellulose) due to the smaller pore size and stronger negative polarity of NC over cellulose (Fig. 2a). In addition, the auto-fluorescence in cellulose paper was lower with NC paper than cellulose paper (Fig. 2b) and the plastic backing of NC paper prevents the vertical flow of coating reagents to the other side of paper [26]. GRGDSPK (RGD-containing peptide) coating was not successful in promoting endothelial cell adhesion. Collagen coating, on the other hand, was much more successful in accommodating endothelial cell adhesion, presumably due to its rigorous coating over paper fibers compared to GRGDSPK (Fig. 2c) as well as collagen’s better representation of natural ECM [27]. Consequently, collagen-coated NC paper was chosen as the optimum substrate for the vascular endothelial chip model. Even though endothelial cells’ migration was observed in other collagen-based scaffold in the previous study [28], the major benefit of collagen-coated NC paper is the flexibility of paper that can be hole-punched and bended, which can be controlled by an inexpensive servo motor and Arduino microcontroller, to generate the shear flow. These cannot be done with collagen or any other gel-based systems [29, 30].

Rat vascular endothelial cells (RVECs) were cultured on collagen-coated NC paper for 24 h, resulting in confluent and healthy adhesion behavior. Additionally, RVECs were patterned only within the peripheral sides of the paper substrate using a PDMS block (Fig. 1), without using any conventional lithography or wax printing techniques. This patterning mimicked pre-existing capillary vessels, and the RVECs were free to migrate in between these two simulated vessels, demonstrated in the following sets of experiments.

The assay time for sufficient cell migration was optimized using these cell-patterned paper chips. The migration of RVECs on the paper chip was induced by VEGF at the center of the unoccupied central area of each paper chip, followed by incubation for 1, 3, or 5 h. Both the number of migrating RVECs and the length of migration were used as measures for migration (Fig. 2d and Fig. 2e). 5 h incubation time illustrated the most successful migration before the detachment of cultured cells from paper surface occurred, and was chosen for the rest of the experiment.

VEGF and S1P were also compared by monitoring the migration of RVECs towards the central area in the chip after 5 h of incubation. VEGF plays significant roles in proliferation, differentiation, sprouting, and migration of endothelial cells during angiogenesis. S1P is known to primarily enhance the migration of immune cells, for example, T- and B-cells, while it can also induce the migration of endothelial cells. Both the number of migrating cells and the length of migration pattern with VEGF were significantly different from those without (*p* < 0.05), and with S1P induction. Since the use of S1P did not result in sufficient angiogenic behavior on the paper chip while VEGF did, VEGF was selected as an optimal chemical stimulant for the rest of the experiments.

### Mechanical induction

**Fig. 3:**
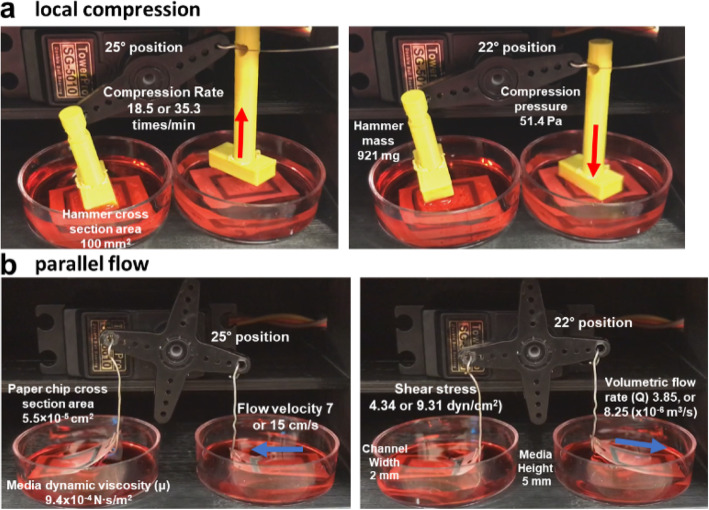
Still images from the video clips showing local compression (**a**) and parallel flow (**b**) applied to the paper chips. Cells are patterned at the top and bottom sides within the black-colored rectangle on a paper chip

Various types and rates of mechanical stimuli were investigated with a single platform developed in this study. Local compression was applied to the paper chip by moving a 3D-printed hammer up-and-down (Fig. 3) but not physically touching the chip surface. Parallel and perpendicular flows were simulated by replacing the 3D-printed hammer with metal wires and lifting the paper up-and-down. This created relative pulsatile flow of the media on top of the cells cultured on the paper chip. Such flows were made in either a parallel or perpendicular direction to the peripherally patterned RVECs (Fig. 1). The rates of the servo motor movement were set to 10 or 15 RPM (100 and 50 ms delay time, respectively), which corresponded to the compression rates of 18.5 or 35.3 times/min or the flow rates of 7 or 15 cm/s. These flow rates correspond with the average arterial flow rate [31].

Compression force delivered to the vascular endothelial cells on the paper chip was calculated using a simple force balance [32] as shown in Eq. 1.

1 $$ \mathrm{Compression}\ \mathrm{force}\ F= ma-\rho Va $$

Hammer mass (*m*) was 921 mg; acceleration (*a*) from the hammer movement was 5 m/s^2^; medium density (*ρ*) at 37 °C was 1 g/cm^3^; total volume of a submerged hammer (*V*) was 150 mm^3^. The resulting compression force (*F*) was 3.86 g·m/s^2^. Compression pressure was calculated using Eq. 2.

2 $$ \mathrm{Compression}\ \mathrm{pressure}\ P=F/A $$

With the hammer area (*A*) of 75 mm^2^ and the compression force (*F)* calculated above, the compression pressure experienced by vascular endothelial cells was 51.4 kg/m·s^2^ = 51.4 Pa (Table 1), which corresponded to the pressure of 68 Pa in vein [32]. Wall shear stress was calculated using Eq. 3 [33].

3 $$ \mathrm{Shear}\ \mathrm{stress}\ \tau =6\mu Q/{H}^2W $$

**Table 1:** Calculation of compression pressure

**Cross section area (mm**^**2**^**)**	75
**Acceleration (m/s**^**2**^**)**	5
**Mass (mg)**	921
**Pressure (Pa)**	51.4

Dynamic viscosity (*μ*) of the media was 9.4 × 10^− 4^ N∙s/m^2^; the volumetric flow rates (*Q*) were calculated as 3.85 × 10^− 6^ m^3^/s (for 7 cm/s flow rate) and 8.25 × 10^− 6^ m^3^/s (for 15 cm/s flow rate). The height of media from the paper surface, 5 mm, was used for *H* and the channel width on the paper chip, 2 mm, was used for *W*. The resulting wall shear stresses were 4.34 dyn/cm^2^ for 7 cm/s flow rate and 9.31 dyn/cm^2^ for 15 cm/s flow rate, respectively (Table 2), which also corresponded with the average shear stress of 4–30 dyn/cm^2^ within arteries [34]. The effect of paper bending to the cells can also be considered. The surface stress and stiffness resulting from the bended paper chip was negligible according to the thin plate theory on the stiffness of thin sheet (paper chip in our case) that is independent of its bending state within the framework of linear elasticity [35].

**Table 2:** Calculation of shear stress

**Servo motor movement rate (RPM)**	10	15
**Flow velocity (m/s)**	0.07	0.15
**Cross section area (m**^**2**^**)**	5.5 × 10^−5^	5.5 × 10^− 5^
**Volumetric flow rate, Q (m**^**3**^**/s)**	3.85 × 10^−6^	8.25 × 10^− 6^
**Dynamic viscosity, μ (N∙s/m**^**2**^**)**	9.4 × 10^−4^	9.4 × 10^−4^
**Height, H (m)**	0.005	0.005
**Width, W (m)**	0.002	0.002
**Shear stress, τ (dyn/cm**^**2**^**)**	4.34	9.31

**Fig. 4:**
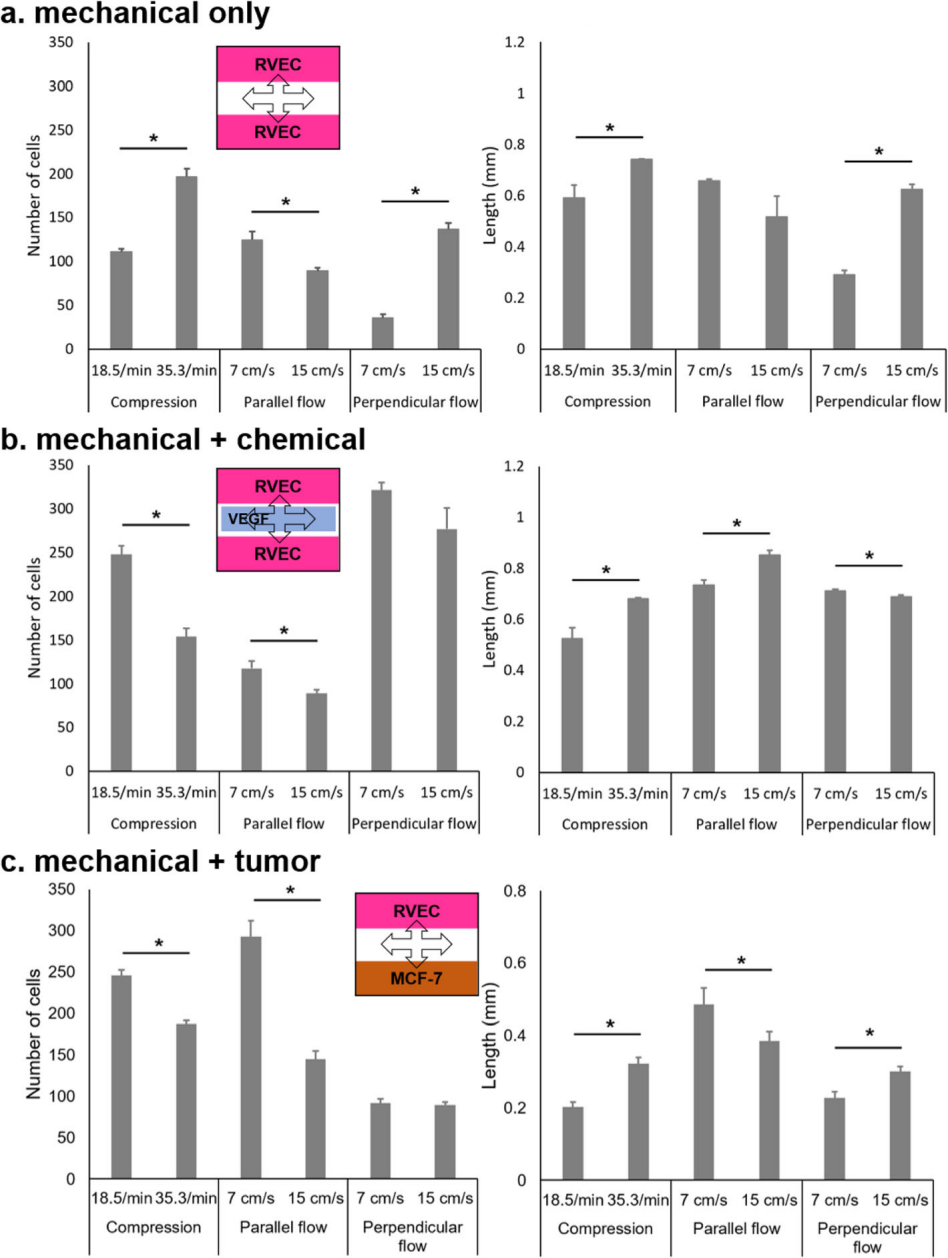
Average numbers of RVECs in FOV and average lengths of RVEC migration on the paper chips with **a** mechanical stimuli, **b** mechanical and chemical stimuli (VEGF), and **c** mechanical stimuli and tumor induction, where one peripheral channel was replaced with MCF7 (breast cancer cells). Averages of 3 experiments, each from 6 different images of a different paper chip. Error bars represent standard errors. * represents statistical difference with *p* < 0.05

All three types of mechanical stimuli (local compression, parallel flow, and perpendicular flow) effectively induced the migration of RVECs toward the unoccupied central channel on the paper chip without any chemical induction, as shown in Fig. 4a and Fig. S3 and Table S1 in Additional file 3. With the exception of the perpendicular flow at a low flow rate of 7 cm/s, the average numbers of migrating cells ranged from 89 to 197 with mechanical stimuli and 31 with static condition, all statistically different with *p* < 0.05, and the average lengths of migration ranged from 0.516 to 0.741 mm with mechanical stimuli and 0.089 with static condition, again all statistically different with *p* < 0.05 (Table S1 in Additional file 3). The highest number of migrating cells and length of migration were 197 and 0.741 mm, respectively, which are comparable to those with VEGF (chemical induction), 215 and 0.895, respectively. These results correspond to the study of Hsu et al. on the effects of different flow patterns to vascular endothelial cell migration without any chemical induction [36].

The higher compression rate (35.3 times/min) induced a significantly higher number of migrating cells, 197 cells, than 111 cells with the lower compression rate (18.5 times/min) (*p* < 0.05); however, there was a statistically insignificant increase in migration length, 0.741 mm vs. 0.59 mm (*p* > 0.05). With the perpendicular flow, the faster flow rate (15 cm/s) significantly improved (*p* < 0.05) both the number of migrating cells and the length, 136 cells and 0.63 mm length, than those with the slower flow rate (7 cm/s), 36 cells and 0.29 mm length. In contrast, the parallel flow rate did not induce statistical differences (*p* > 0.05) in the number of migrating cells and the migration length: 125 cells and 0.671 mm migration length with 15 cm/s and 89 cells and 0.52 mm migration length with 7 cm/s.

### Mechanical and chemical induction

The most physiologically relevant method of induction would be combined induction of mechanical and chemical stimuli. For chemical induction, VEGF was chosen considering its major role in increasing vascular permeability and cellular migration [37]. VEGFR-2, a major receptor mediating most angiogenic functions, is upregulated in endothelial cells when seeded in a 3D collagen matrix, increasing the endothelial cells’ sensitivity to VEGF [38]. We hypothesized that the fibrous and porous structures of NC paper with collagen coating could provide a 3D microenvironment similar to the collagen matrix, leading to successful migration of endothelial cells in response to VEGF.

**Fig. 5:**
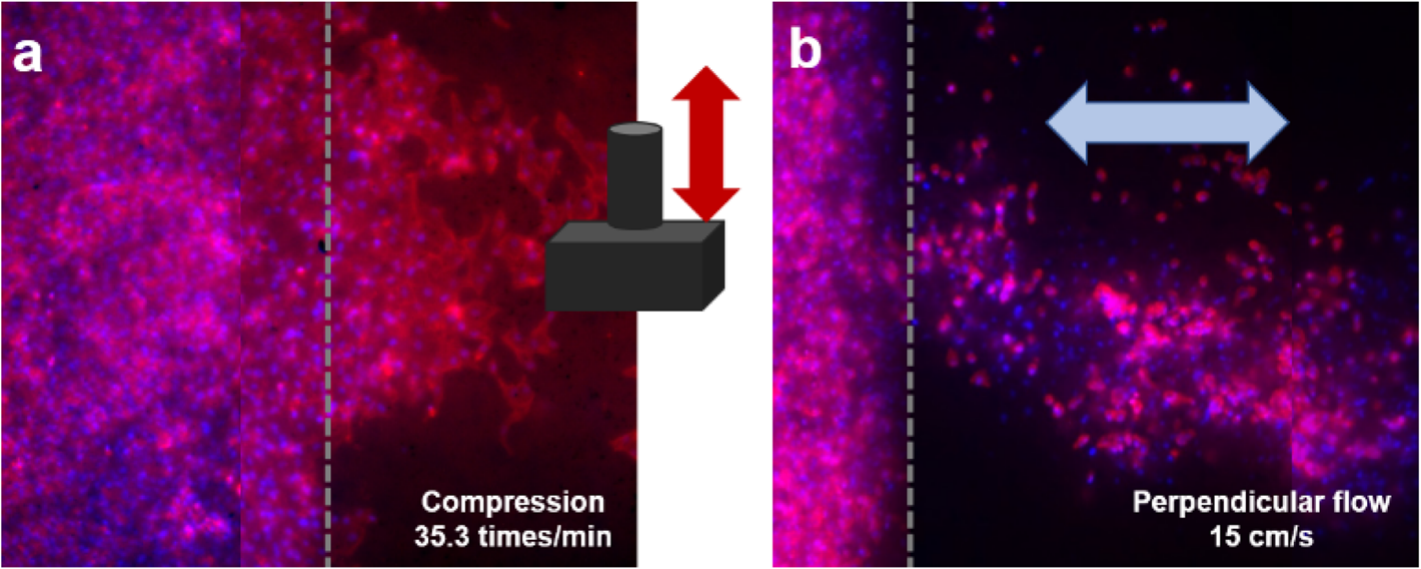
Representative fluorescence images of RVEC migration under the combined chemical (VEGF) and mechanical stimuli: **a** local compression at the rate of 35.3 times/min and **b** perpendicular flow at the rate of 15 cm/s. Grey dashed line represents the border where the cells were initially seeded. A hammer in (**a**) delivers the compression stimuli, and the light blue arrow in (**b**) represents the direction of perpendicular shear flow

Three different mechanical stimuli were applied to the RVEC-patterned NC paper chips, with VEGF added to the central channel area. Representative fluorescence images of RVEC migration in response to the combined mechanical and chemical stimuli are shown in Fig. 5 (other images are available in the supplementary figure in Additional file 3). In general, both the number of migrating cells and the migration length increased to 89–321 cells and 0.525–0.823 mm from 36 to 197 cells and 0.291–0.741 mm without VEGF (89–197 cells and 0.516–0.741 excluding the perpendicular flow at the low flow rate) (Fig. 4a and b, as well as Table S1 in Additional file 3). These numbers are of course higher than 31 cells and 0.089 mm under static condition. These effects of mechanical and chemical induction on RVEC migration are consistent with those of VEGF-induced human umbilical vein endothelial cell migration under 2.09 dyn/cm^2^ shear force [5].

Specifically, significant increases in the number of migrating cells were observed at lower compression rate (111 vs. 248 cells) and at slower perpendicular flow rate (36 vs. 321 cells). A similar trend could be observed in the migration length at a slower perpendicular flow rate (0.29 vs. 0.712 mm length). At a higher compression rate and faster perpendicular flow rate, however, the number of migrating cells and the migration length did not increase further or even decreased compared to those without VEGF. No significant improvement with VEGF was observed for parallel flow. Taken together, the numbers shown in Fig. 4b could potentially be the maximum extent of migration.

### Mechanical and tumor induction

Tumor cells release VEGF to induce migration of endothelial cells and subsequently angiogenesis to provide nutrients and oxygen for their growth and persistence [39]. Such tumor-induced endothelial cell migration was also demonstrated on the developed paper chip and device. MCF7, breast cancer cells, were seeded on one side of the paper chip and RVECs on the other side. RVECs and MCF7s were separately cultured under static conditions. Cells were patterned using the aforementioned PDMS block. After that, the block was removed, and the MCF7 cells were expected to release VEGF to induce migration of the RVECs on the other side of the paper chip. No additional VEGF was added in these experiments. While no migration was observed after 3 h static incubation, RVECs started migrating toward the opposite MCF7 side after 5 h static incubation. These experiments were repeated by adding mechanical stimuli (local compression, parallel flow, and perpendicular flow) to further enhance the migration of RVECs (Fig. 4c). Again, no additional VEGF was added. Overall, the numbers of migrating cells with both tumor induction and mechanical stimuli significantly increased from those with mechanical stimuli only and were comparable to those with combined chemical and mechanical stimuli (Fig. 4a, b and c, as well as Table S1 in Additional file 3). In addition, a higher compression rate and higher flow rate did not substantially increase the number of migrating cells with both tumor induction and mechanical stimuli, similar to the results with combined chemical and mechanical stimuli. The only difference was that the parallel flow was preferred with tumor induction and mechanical stimuli while the perpendicular flow was preferred with chemical and mechanical stimuli. The lengths of migration with tumor induction and mechanical stimuli were very similar to those with chemical and mechanical induction, while the differences were more pronounced with tumor induction (Fig. 4c and Table S1 in Additional file 3).

## Conclusion

The device presented in this work is able to deliver two different mechanical stimuli of local compression and shear flow to an in vitro tissue chip, as well as simultaneous introduction of chemical stimuli and tumor induction. It demonstrates the advantages of both gel-based 3D cell culture models and silicone-based microfluidic tissue models. Vascular endothelial cells’ migration was demonstrated as an example of dynamic tissue development in response to mechanical stimuli. This aim was achieved by utilizing paper-based cell culture that was low cost, easy to fabricate, porous, and most importantly flexible. Mechanical stimuli were delivered in a programmed manner using an Arduino microcontroller. The cost for equipment and supplies is also low, with ~US$17 for an Arduino microcontroller, ~US$12 for a servo motor, and the total equipment/supplies cost < US$50 at the time of writing. The device is also small enough to be placed in the shelves of typical CO_2_ incubators with its own battery supply. Most importantly, large number of experimental conditions can be evaluated using this single device, e.g., many different combinations of mechanical and chemical stimuli (including stretch/strain through bending the paper at much larger degrees) as well as tumor induction, large number of rate combinations for mechanical stimuli, and unrestricted growth of tissue structures (not limited by pre-defined channels). With these possibilities, the proposed device can be used for high-throughput studies and big data analyses, which can be potentially useful in screening and optimizing drugs and therapeutic strategies.

## Methods

### Device housing

**Fig. 6:**
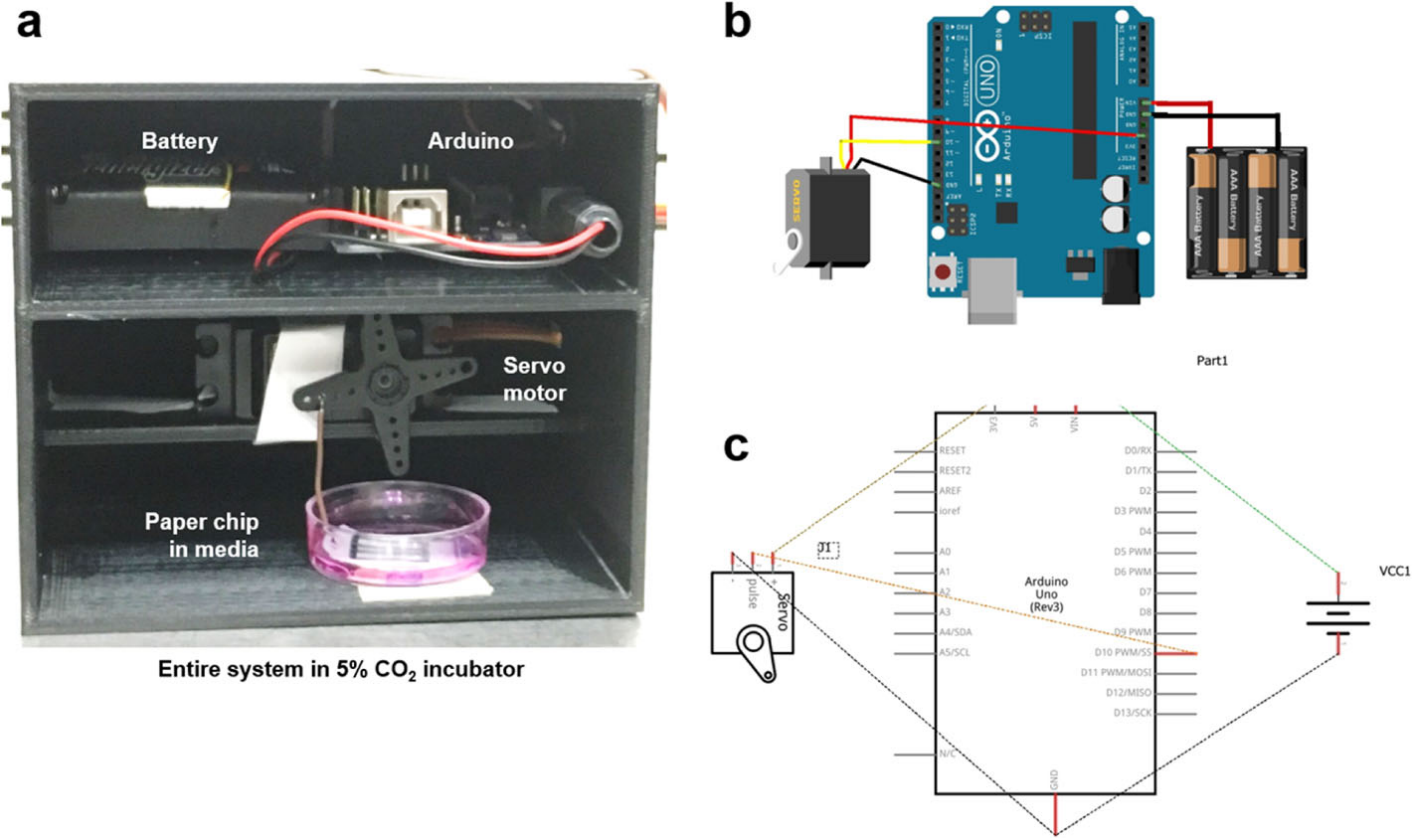
The device housing (**a**) incorporates batteries, Arduino microcontroller, and a servo motor (**b**), along with the paper chip submerged in media. Detail pin configurations are shown in (**c**)

The housing of the device was designed and 3D-printed with acrylonitrile-butadiene-styrene (ABS) co-polymer using MakerBot Z18 (MakerBot Industries, Brooklyn, NY, USA), with the total dimension of 11.7 cm × 9.7 cm × 9.7 cm. There were three shelves in the housing as shown in Fig. 6a; four AA batteries and an Arduino Uno microcontroller in the top shelf (configurations for pin connections shown in Fig. 6b and Fig. 6c), a servo motor with 38 mm arm length and 180° rotation (Kookye MG 995; Pinetree Electronics Ltd., Richmond, BC, Canada) in the middle shelf, and the paper chip submerged in culture media within petri dishes in the bottom shelf.

### Optimization of paper and coating materials

To optimize cell adhesion on paper, paper types of cellulose (GE Healthcare, Maidstone, Kent, UK) vs. nitrocellulose (NC; EMD Millipore, Hayward, CA, USA) were tested and compared. In addition, coatings of RGD-containing peptide (GRDGSPK; 50 μg/mL; AnaSpec Inc., Fremont, CA, USA) vs. collagen (type I rat tail; 50 μg/mL; BD, Franklin Lakes, NJ, USA) were tested to compare the difference between cell-binding peptide vs. whole protein. The following paper-coating type combinations were tested: RGD-cellulose, collagen-cellulose, RGD-NC, collagen-NC, cellulose only, nitrocellulose only, and standard 96-well tissue culture plate (TCP; Corning Inc., Corning, NY, USA). Paper substrates were hole-punched into 5-mm diameter circles, individualized into a 96-well plate, and UV sterilized prior to experiments. 100 μL of rat vascular endothelial cells at 5000 cells/mL (RVECs; ATCS, Manassas, VA, USA) were added to each paper-coating combination, incubated for 1 h, then washed twice with phosphate buffer saline (PBS; Sigma-Aldrich, St. Louis, MO, USA). Fluorescence images of each paper substrate were collected using a benchtop fluorescence microscope (Nikon Eclipse TS100, Minato, Tokyo, Japan) with UV filter attachments (A.G. Heinze, Lake Forest, CA, USA). Cells were stained with DAPI (UV excitation and blue emission) to count the number of cells in the field of view (FOV) using ImageJ software (National Institutes of Health, Bethesda, MD, USA). Details of fluorescence imaging are described in the later section of Fluorescence imaging.

### Cell culture

Rat vascular endothelial cells (RVECs; ATCC, Manassas, VA, USA) were maintained in Dulbecco’s Modified Eagle Medium (DMEM; Corning) supplemented with 10% v/v fetal bovine serum (FBS; Fisher Scientific, Pittsburgh, PA, USA), 0.2% v/v of 250 μg/mL Amphotericin B (GE Healthcare), and 0.1% v/v of 50 mg/mL Gentamycin sulfate on T-75 cell culture flasks (Greiner Bio-One, Monroe, NC, USA) under static conditions at 37 °C with 5% CO_2_ (HERAcell 150i; Thermo Scientific, Waltham, MA, USA) until they reached 90% confluency. They were re-suspended at a final concentration of 2 × 10^6^ cells/mL.

MCF7 were maintained and cultured in the same manner as culturing RVECs, where DMEM was replaced with Eagle’s Minimum Essential Medium (Corning) and 0.01 mg/mL human recombinant insulin (Sigma-Aldrich) was additionally added. MCF7 was patterned in the same manner as patterning RVECs.

### Cell patterning

Prior to cell patterning, NC paper (thickness = 2 mm; average pore size = 14.53 μm) was cut into 11 mm wide and 15 mm long pieces. 0.1 mL of 50 μg/mL collagen solution (= 5 μg of collagen) was added to cover the entire surface of NC paper and left for 1 h to allow ubiquitous coating, followed by washing twice with PBS. The type (NC) and coating (collagen) of paper were determined from the experiments described in the previous section. Fluorescamine protein assay (Thermo Fisher Scientific, Waltham, MA, USA) was used to verify the collagen coating on NC paper following the vendor’s protocol. A 15 mm × 5 mm × 5 mm PDMS block was placed on the paper chip and held by two 12 mm × 4 mm × 2 mm neodymium block magnets (one placed on PDMS block and one underneath NC paper), exposing two rectangular areas of 3 mm wide and 15 mm long each (Fig. 1). 10 μL of the cell solution was seeded on these 3 mm-wide peripheral sides of the paper chip. Cells were allowed to anchor on the surface for 15 min, 3 mL of endothelial growth media was added to cover the paper, and the cells were cultured under static condition for 24 h, until the cells monolayerly covered the peripheral channel to mimic the monolayer of vascular endothelial cells surrounding the blood vessel in vivo.

### Optimization of assay time and chemical stimuli

Following 24 h static cell culture, the collagen-coated NC paper chips were washed twice with DPBS. Either 0.1% v/v of 50 ng/mL vascular endothelial growth factor (VEGF; Invitrogen, Carlsbad, CA, USA) or 0.1% v/v of 50 ng/mL sphingosine-1-phosphate (S1P; Echelon Biosciences Inc., Salt Lake City, UT, USA) was loaded to the central areas of the paper chips as chemical stimuli. The paper chips were dipped into 3 mL endothelial growth media in a Petri dish as shown in Fig. 6, and the cells were incubated under static condition at 37 °C within a 5% CO_2_ incubator over the time course (1, 3, and 5 h). After incubation, RVECs on each paper chip were imaged using a benchtop fluorescence microscope, where the cells’ nuclei were stained with DAPI and the cells’ actin filaments were stained with TRITC-phalloidin. These two images were stacked onto each other to represent the entire cell images. Since the entire migration pattern cannot be captured in a single frame, multiple images were captured and connected by using the overlap area of each individual image to represent the whole length and pattern of cell migration. Red arrow symbols were added to all images to indicate the points where the images were connected. The grey dotted lines represent the border of the channel where the cells were initially seeded. To measure the length of migrating cells in 2D, the straight line was used to measure the length in pixels then converted to the length in millimeter scale.

### Mechanical induction

The paper chips with patterned cells were exposed to three different types of mechanical stimuli (Fig. 1). Local compression was introduced by placing the paper chips underneath a plastic hammer. These hammers were designed using SolidWorks software (SolidWorks Corp., Waltham, MA, USA), 3D-printed with ABS co-polymer using Zortrax M200 (Zortrax, Olsztyn, Poland), and subsequently sterilized by dipping in 70% ethanol and dried under UV light for 30 min prior to cell experiment. The paper chip was cut into the size that corresponds to a diameter of petri dish in order to allow the unidirectional movement of paper and distribution of delivered local compression along the length of patterned RVECs. Parallel and perpendicular flows were introduced by connecting the metal wire (sterilized following the above-mentioned protocol) that moved up and down through the hole-punched on one end of the NC paper chips to create relative flow of media over the chips.

The hammers and metal wires were connected to a 180-degree range servo motor, which was connected to and controlled by an Arduino Uno microcontroller (Fig. 6). The servo motor was programmed to rotate over a very narrow range of angles, from 22° to 25°, to gently tap the paper chip surface but not physically touching it for delivering local compression, or to lift the paper chip up and down over a short distance for delivering relative share flow, all at the rotation rates of 10 or 15 RPM. The video clips of local compression and shear flow were recorded (MOV files are included in the supplementary information as Additional files 1 and 2; still images are shown in Fig. 3) and then used to calculate the compression rates (18.5 times/min or 35.3 times/min) and the relative flow rates (7 cm/s or 15 cm/s). This setup was maintained for 5 h under 37 °C and 5% CO_2_.

### Mechanical stimuli added with chemical stimuli or tumor induction

In addition to the mechanical stimuli, chemical stimuli and tumor induction were also applied. As previously described in the Optimization of assay time and chemical stimuli section, VEGF was pre-loaded to the central areas of the paper chips as the optimum chemical stimuli, optimized from the experiments described in the same section. For tumor induction, one side of the channel was seeded with MCF7, a human breast cancer cell line, rather than seeding both channels with RVECs. MCF7 were maintained, cultured, and patterned in the same manner as culturing and patterning RVECs (differences were addressed in the supplementary information – Additional file 3)*.*

### Fluorescence imaging

Waste media was removed from the paper chips, which were then fixed with a 4% solution of paraformaldehyde (Affymetrix, Santa Clara, CA, USA) for 15 min. Paraformaldehyde was removed and paper chips were rinsed twice with washing buffer solution (1X PBS with 0.05% Tween-20). Cell membranes were perforated with 0.1% Triton-X-100 (Fisher Scientific) for 5 min. Triton-X was removed and paper chips were rinsed twice with washing buffer solution. Paper chips were treated with blocking buffer solution (1% bovine serum albumin - BSA in 1X PBS) and then were incubated for 30 min. The cells’ nuclei were stained with DAPI and actin filaments with TRITC-conjugated phalloidin (EMD Millipore, Burlington, MA, USA). Fluorescence images were collected using the ISCapture software on a personal computer connected to a benchtop fluorescence microscope (Nikon Eclipse TS100, Minato, Tokyo, Japan) with UV and TRITC filter attachments (A.G. Heinze, Lake Forest, CA, USA). Since the entire migration pattern cannot be captured in a single frame, multiple images were captured and connected by using the overlap area of each individual image to represent the whole length and pattern of cell migration. Red arrow symbols were added to all images to indicate the points where the images were connected. The grey dotted lines represent the border of the channel where the cells were initially seeded.

The greyscale images were taken from each filter cube and transferred to ImageJ software (National Institutes of Health, Bethesda, MD, USA). The pseudo-colors were then added: blue to DAPI (cells’ nuclei) and red to TRITC-phalloidin (cells’ actin filaments). These two images were stacked onto each other to represent the entire cell images. To measure the length of sprouting cells in 2D, the straight line was used to measure the length in pixel then converted to the length in millimeter.

### Statistical analyses

All data were derived from at least three replicates, each using a different paper chip with seeded cells. Statistical analyses were performed using analyses of variance (ANOVA). Differences at *p* < 0.05 were considered statistically significant.

## Supplementary information

**Additional file 3. **Supplementary figures and table. **Fig. S1.** Supplementary figures for optimization of paper and coating. **Fig. S2.** Supplementary figures for assay time optimization and chemical induction. **Fig. S3.** Supplementary figures for mechanical induction. **Fig. S4.** Supplementary figures for mechanical plus chemical induction. **Fig. S5.** Supplementary figures for tumor induction. **Table S1.** The number of migrating cells and length of the migrating pattern for each figure.

## Data Availability

Movie clips of device operation are available in the Additional files 1 and 2. Fluorescence microscopic images used to generate Figs. 2, 3 and 4 are available in the Additional file 3. Additional data can be requested to the authors upon reasonable request.
