# Supplementary material for: Paper-based in vitro tissue chip for delivering programmed mechanical stimuli of local compression and shear flow
Source: J Biol Eng. 2020 Jul 28;14:20. doi: 10.1186/s13036-020-00242-5 (PMC7385864; doi:10.1186/s13036-020-00242-5)
Supplement: Supplementary file 3 — Additional file 3. Supplementary figures and table. Fig. S1. Supplementary figures for optimization of paper and coating. Fig. S2. Supplementary figures for assay time optimization and chemical induction. Fig. S3. Supplementary figures for mechanical induction. Fig. S4. Supplementary figures for mechanical plus chemical induction. Fig. S5. Supplementary figures for tumor induction. Table S1. The number of migrating cells and length of the migrating pattern for each figure. [file 13036_2020_242_MOESM3_ESM.pdf]

SUPPLEMENTARY INFORMATION

Paper-based *in vitro* tissue chip for delivering programmed mechanical stimuli of local compression and shear flow

Kattika Kaarj, Marianne Madias, Patarajarin Akarapipad, Soohee Cho, and Jeong-Yeol Yoon

Additional File 3. Supplementary Figures and Table

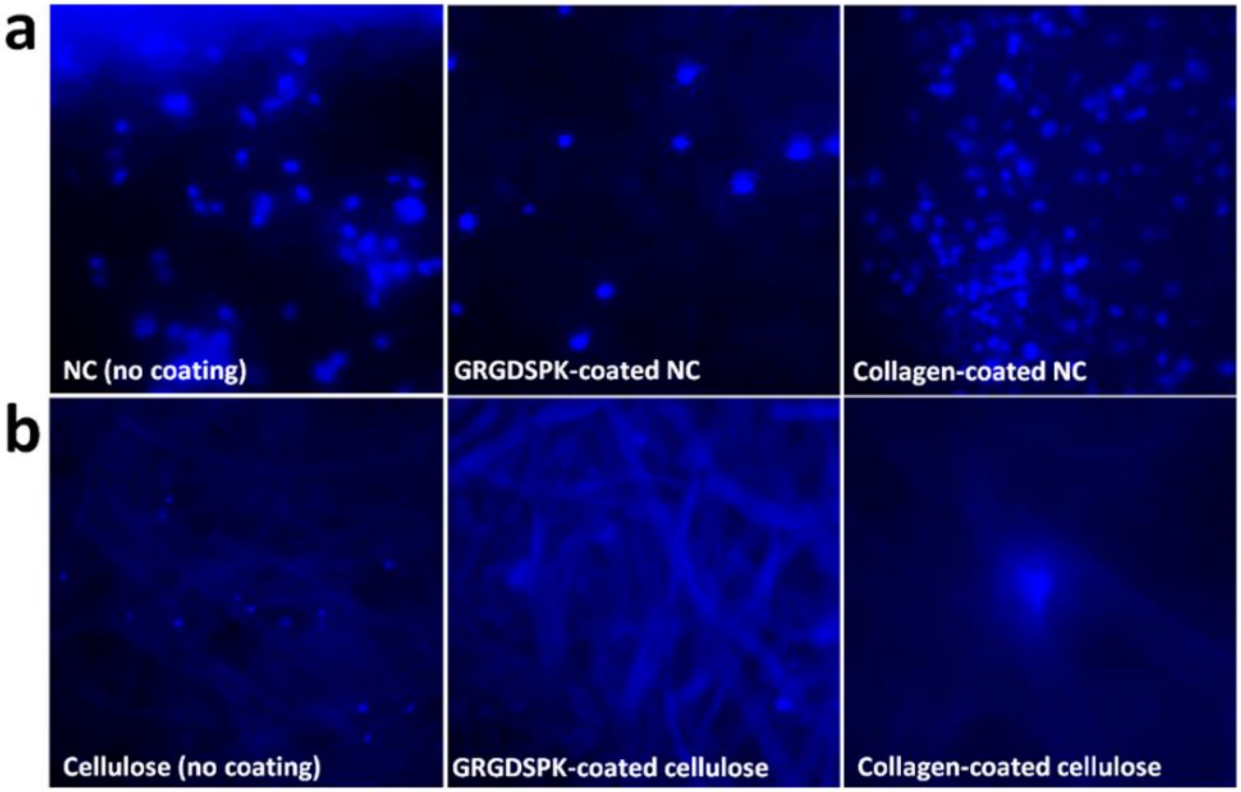

Fig. S1. Optimization of paper and coating. Fluorescent images of DAPI-stained RVECs on various paper types and coatings after 24 h culture. (a) NC with no coating, GRGDSPK-coating, and collagen-coating. 10X objectives were used. (b) Cellulose with no coating, GRGDSPK-coating, and collagen coating. 10X (no coating) and 40X (GRGDSPK and collagen coating) objectives were used (higher magnification was necessary due to auto-fluorescence of cellulose fibers).

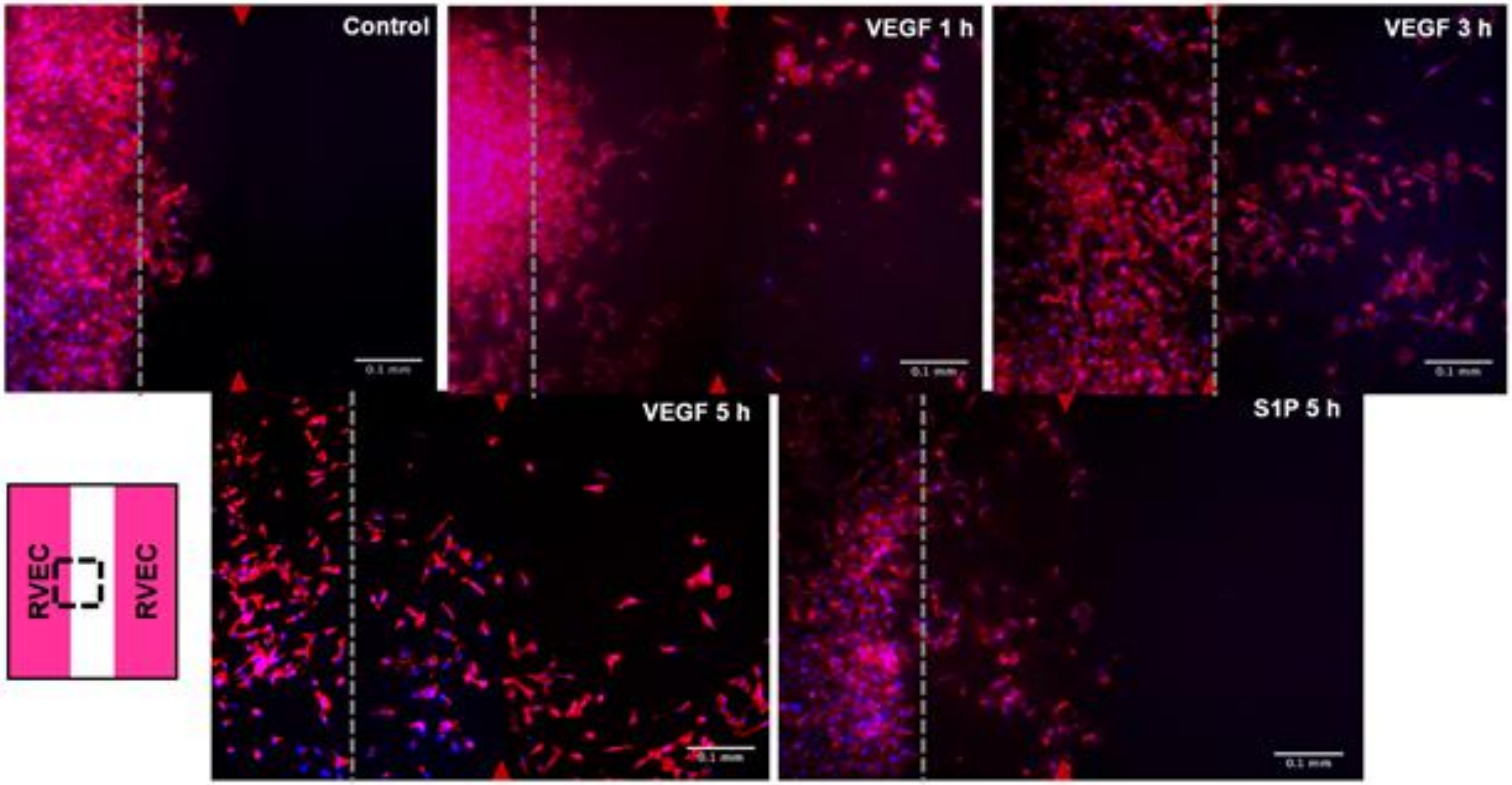

Fig. S2. Assay time optimization and chemical induction. The VEGF induced endothelial cell migration over the time course of 1, 3 and 5 h under the static condition. VEGF and S1P induced cell migration at 5 h under static condition. RVECs were stained with DAPI and TRITC-phalloidin, and the number of migrating cells and the length of migration pattern were evaluated to quantify the extent of vessel sprouting.

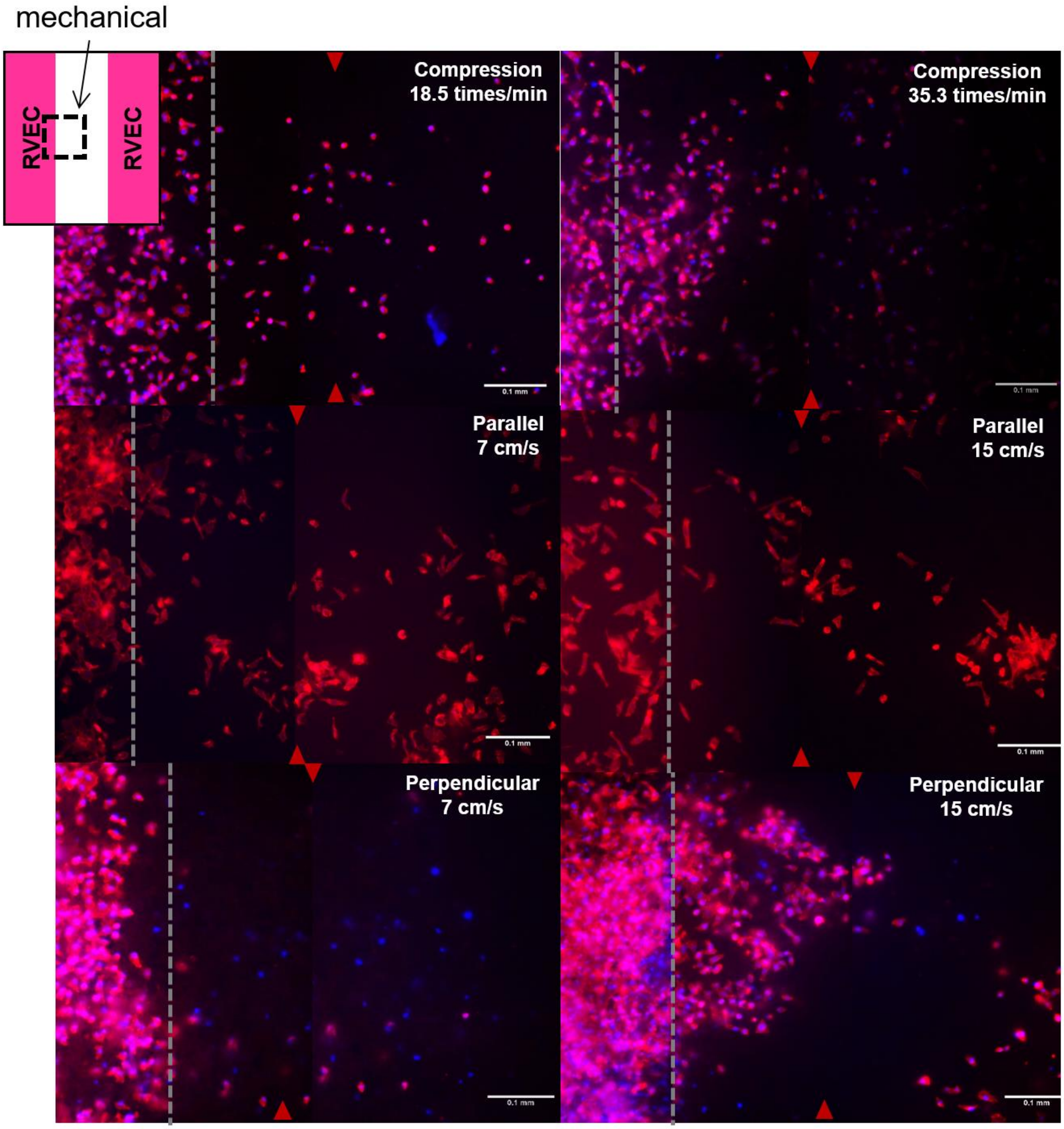

Fig. S3. Mechanical induction. Fluorescence images of RVECs' migration under the local compression at the rate of 18.5 times/min and 35.3 times/min, the parallel flow at the rate of 7 cm/s and 15 cm/s, and the perpendicular flow at the rate of 7 cm/s and 15 cm/s. All images are representative results from at least three different experiments, each time using a different paper model with stamped cells.

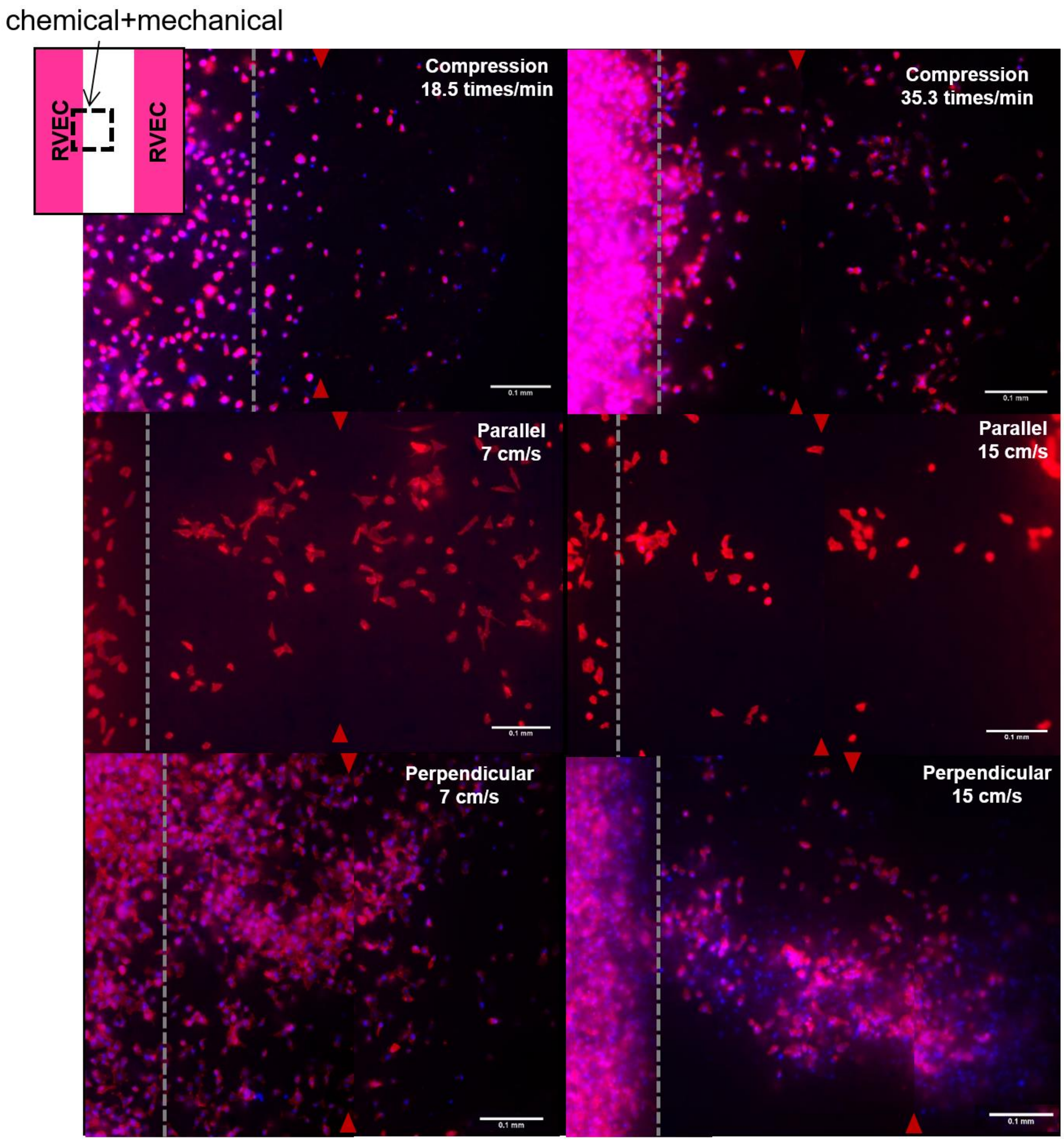

Fig. S4. Mechanical plus chemical induction. Identical experiments with those shown in Fig. S3, with the addition of VEGF.

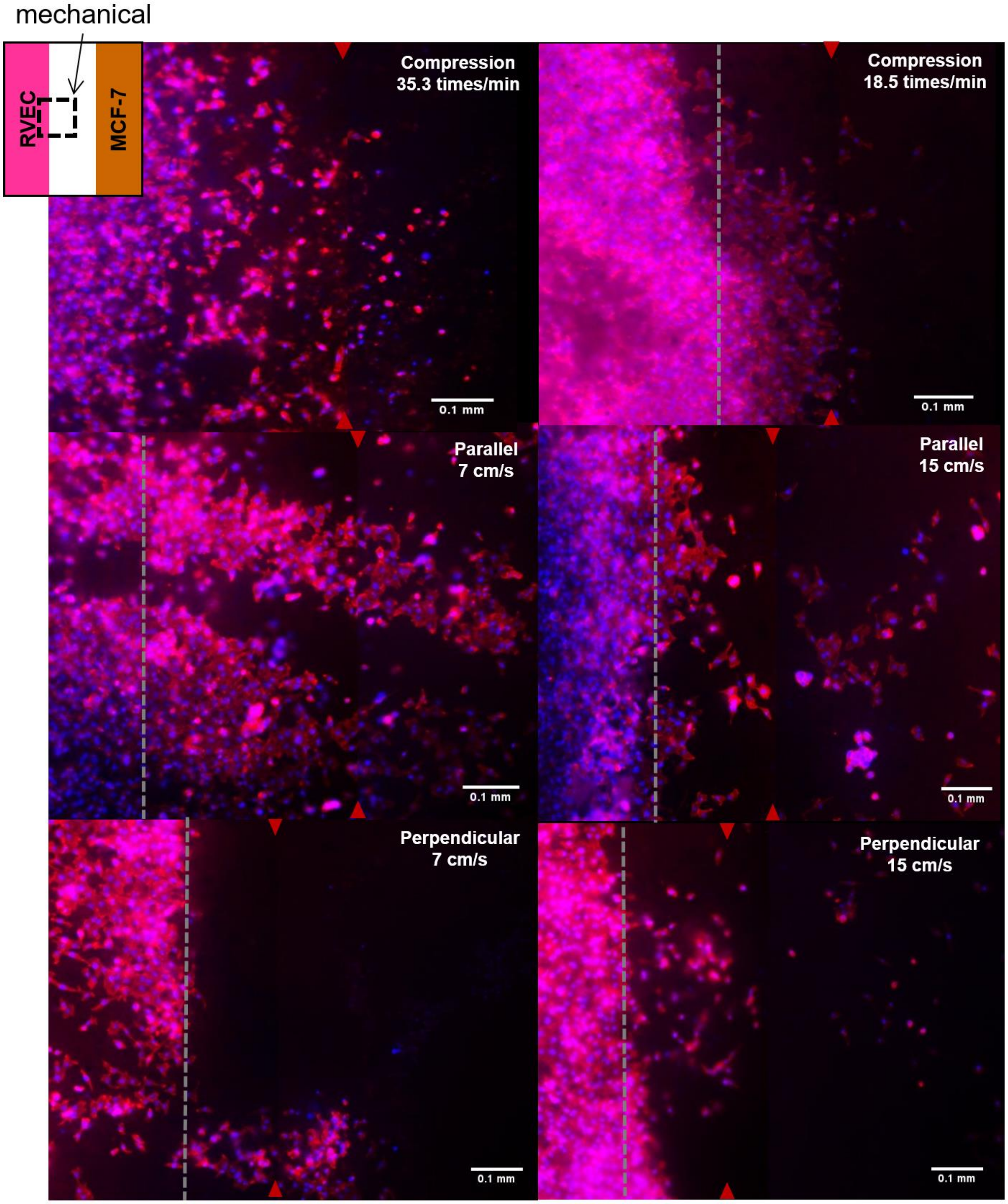

Fig. S5. Tumor induction. The paper models were stamped with RVECs on one side and MCF7 (breast cancer cells) on the other. Identical experiments with those shown in Fig. S3, while using RVEC-MCF7 combination. VEGF was not added.

Table S1. The number of migrating cells and length of the migrating pattern for each figure.

Figure 2. Assay time and effect of chemical induction on cell migration.

Experimental condition	Incubation time	Number of migrating cells	Standard error	Length of migrating pattern (mm)	Standard error
Control	5 h	31	3.5	0.089	0.013
VEGF	1 h	135	10.4	0.237	0.020
	3 h	167	9.1	0.401	0.007
	5 h	215	9.2	0.895	0.162
Experimental condition	Incubation time	Number of migrating cells	Standard error	Length of migrating pattern (mm)	Standard error
Control	5 h	31	3.5	0.089	0.013
VEGF	5 h	215	9.2	0.895	0.162
S1P	5 h	62	3.0	0.233	0.003

Figure 4a. Effect of only mechanical induction on cell migration.

Mechanical stimuli	Rate	Number of migrating cells	Standard error	Length of migrating pattern (mm)	Standard error
Compression	18.5 times/min	111	3.6	0.590	0.051
	35.3 times/min	197	9.1	0.741	0.002
Parallel flow	7 cm/s	125	9.2	0.671	0.029
	15 cm/s	89	3.5	0.516	0.080
Perpendicular flow	7 cm/s	36	4.0	0.291	0.018
	15 cm/s	136	7.8	0.625	0.017

Figure 4b. Effect of both mechanical and chemical induction on cell migration.

Mechanical stimuli	Rate	Number of migrating cells	Standard error	Length of migrating pattern (mm)	Standard error
Compression	18.5 times/min	248	9.5	0.525	0.042
	35.3 times/min	154	9.6	0.681	0.003
Parallel flow	7 cm/s	118	8.1	0.734	0.019
	15 cm/s	89	4.7	0.853	0.016
Perpendicular flow	7 cm/s	321	9.1	0.712	0.006
	15 cm/s	277	24.4	0.689	0.007

Figure 4c. Effect of tumor induced cell migration under mechanical stimuli.

Mechanical stimuli	Rate	Number of migrating cells	Standard error	Length of migrating pattern (mm)	Standard error
Compression	18.5 times/min	246	7.0	0.203	0.013
	35.3 times/min	187	4.5	0.323	0.017
Parallel flow	7 cm/s	293	19.3	0.486	0.045
	15 cm/s	145	9.6	0.385	0.026
Perpendicular flow	7 cm/s	92	5.0	0.229	0.017
	15 cm/s	89	3.5	0.301	0.014

Note. Three different chips were tested. 6 images were acquired from a single chip, each at different location. Standard deviations can be calculated by multiplying the standard errors by 4.24.
